# An Approach to Minimize Tumour Proliferation by Reducing the Formation of Components for Cell Membrane

**DOI:** 10.3390/molecules27092735

**Published:** 2022-04-24

**Authors:** Inmaculada de Dios-Pérez, Álvaro González-Garcinuño, Eva María Martín del Valle

**Affiliations:** Department of Chemical Engineering, University of Salamanca, 37008 Salamanca, Spain; inmadedip@usal.es (I.d.D.-P.); alvaro_gonzalez@usal.es (Á.G.-G.)

**Keywords:** farnesol, phosphatidylcholine, phosphocholine cytidylyltransferase, cyclodextrins, sulfobutylated β-cyclodextrin

## Abstract

Isoprenoids are natural compounds essential for a great number of cellular functions. One of them is farnesol (FOH), which can reduce cell proliferation, but its low solubility in aqueous solvents limits its possible clinical use as a pharmacological tool. One alternative is the use of cyclodextrins (CDs) which house hydrophobic molecules forming inclusion complexes. To assess FOH potential application in anticancer treatments, Sulfobutylated β-cyclodextrin Sodium Salt (SBE-β-CD) was selected, due to it has high solubility, approbation by the FDA, and numerous studies that ensure its safety to be administered parenterally or orally without nephrotoxicity associated. The therapeutic action of farnesol and complex were studied in different carcinoma cells, compared with a normal cell line. Farnesol showed selectivity, affecting the viability of colon and liver cancer cells more than in breast cancer cells and fibroblasts. All cells suffered apoptosis after being treated with 150 μM of free FOH, but the complex reduced their cell viability between 50 and 75%. Similar results were obtained for both types of isomers, and the addition of phosphatidylcholine reverses this effect. Finally, cell cycle analysis corroborates the action of FOH as inducer of a G0/G1 phase; when the cells were treated using the complex form, this viability was reduced, reaching 50% in the case of colon and liver, 60% in fibroblasts, and only 75% in breast cancer.

## 1. Introduction

Cancer is considered one of the main social and economic concerns of the public health systems. It is currently the second most common cause of human mortality in the world after cardiovascular diseases. In 2020, cancer was responsible for approximately 10 million deaths worldwide [1]. Cytotoxic drugs are the most widely used cancer treatment, but their action is not selective, causing serious side effects. For this reason, the development of new treatments (with selective action) using cytostatic drugs is being studied. The main drawback of using cytostatic drugs is that they are less effective than traditional cytotoxic drugs, requiring longer treatments. However, prolonged use of cytostatics can have adverse effects [2].

**Figure 1:**
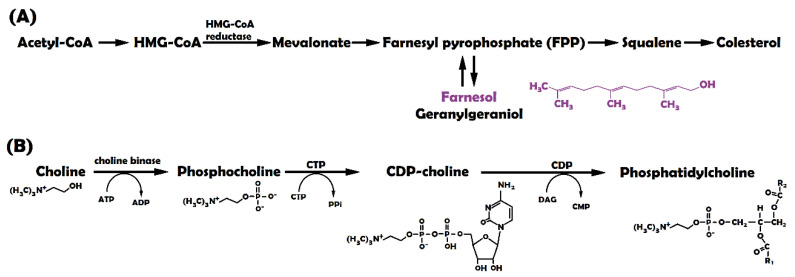
(**A**) Farnesol formation in mevalonate pathway [5]; (**B**) Phosphatidylcholine formation.

Isoprenoids are natural compounds produced from a common precursor, mevalonate (a diagram of the isoprenoid pathway is summarized in Figure 1A). All isoprenoids are essential for numerous cellular functions, including cell signalling, lipid and protein synthesis, maintenance of membrane integrity, cell proliferation, cell cycle arrest, and apoptosis [3]. During recent years, there has been a growing interest in their potential clinical applications due to their multiple purposes in different treatments, including antifungal, antibacterial, antiviral, antitumour, antiparasitic, hypoglycaemic, anti-inflammatory, and analgesic use [4,5,6]. Moreover, some authors have identified that isoprenoids with hydroxy groups possess more anticancer activity than the corresponding compounds with hydrocarbon groups [7,8]. Different authors have suggested the possibility of using farnesol (FOH), an isoprenoid with a hydroxy group, as an inducer of G0/G1 cell cycle arrest and apoptosis in a variety of carcinoma cell types. These studies indicate that FOH can reduce cell proliferation, with half maximal inhibitory concentration (IC50) ranging from 25 to 250 µM [3,5,9,10,11,12]. 

The first paper in which FOH showed selective toxicity was published by Adany et al. in 1994 [13]. According to the authors, tumour cells are generally more sensitive to farnesol-induced growth inhibition than normal cells. After 48 h of treatment using 10 µM FOH, the viability of neoplastic cell lines reduced up to 10%, and when the dosage reached 45 µM, all tumour cells suffered apoptosis [10,13,14]. Other articles have studied the mechanism of action of FOH, suggesting that it depends on different signalling pathways [4,5,6].

One of the most studied signalling pathways is related to cell membrane formation. The rapid proliferation of cancer cells requires an accelerated cell membrane formation. Several studies report that exogenous FOH modulates subcellular localization and activity of phosphocholine cytidylyltransferase (CTP), a rate-limiting enzyme in the cytidine diphosphate-choline (CDP-choline) pathway (Figure 1B). CTP participates in the biosynthesis of phosphatidylcholine (PC), which is one of the major membrane lipids (accounting for 50% of the total phospholipids in mammalian cells) and a precursor of lipid second messengers [15,16,17,18,19]. In this way, Voziyan et al. [20] studied the effect of 20 μM FOH on leukemic cells (CEM-C1). When FOH is added, CTP rapidly translocates to the nuclear envelope (NE) resulting in nucleoplasmic reticulum proliferation and a transient increase in CTP activity. CTP is subsequently exported from the nucleus to the cytoplasm, disrupting PC synthesis. In addition, the activation of caspases during farnesol-induced apoptosis results in CTP cleavage [5].

Other effects that have been reported for farnesol activity in tumour cells include: reduction in HMG-CoA reductase [21,22]; activation of the extracellular signal-regulated kinase (ERK1/2) and p38 mitogen-activated protein kinase (MAPK) [4,9]; translocation of protein kinase C (PKC) from membrane fraction to cytosol [14]; inhibition of Janus-like kinase (JAK1 and JAK2) and induction of proto-oncogene tyrosine-protein kinase Src (c-Src kinases) activation by the inhibition of a constitutive signal transducer and activator of transcription 3 (STAT3) [23]; an increase in the expression of the cyclin-dependent kinase (Cdk) inhibitors p21 Cip1 and p27 Kip1, and a reduction in levels of cyclin A, cyclin B1, and protein Cdk2 [5,12]; and, finally, activation of caspases 3, 6, 7, and 9 [4,11,24]. 

Further FOH effects have been observed, but their influence on mammalian cell apoptosis is unknown. For example, FOH increases the levels of reactive oxygen species (ROS), activates the nuclear farnesoid X receptor (FXR), inhibits thyroid hormone receptor beta (THRβ) mediated transcriptional activation, and inhibits Ca^2+^ signalling by blocking the plasma membrane channels [5,25]. In addition, FOH may inhibit cholesterol biosynthesis, causing a reduction in the proliferation of tumour cells, as they require increased levels of cholesterol [9].

One drawback of the previously mentioned studies is the lack of information concerning the FOH isomers used (the chemical structure of FOH isomers is shown in Supplementary Material S1, Figure S1). Some studies do not mention which isomers have been used [21,26,27,28], while others only use trans-trans isomers (*E*,*E*-FOH) [25,29,30]. Peroxisome proliferator-activated receptors (PPAR) are nuclear receptors with multiple cellular functions. *E*,*E*-FOH has been reported to destabilize the transcriptional activation of PPAR-response elements (PPRE), while *Z*,*Z* isomers remain inactive [5,8]. However, *E*,*E*-FOH is five times more expensive than the mix of isomers. Thus, the application of these pure isomers would increase the cost of treatment and limit its clinical uses [21,30].

Although FOH acts as an anticancer agent, its low solubility in aqueous solvents (7.65 × 10^−6^ M in water) [31] narrows its possible clinical applications. Most isoprenoids are hydrophobic compounds, so organic solvents such as ethanol or dimethyl sulfoxide (DMSO) are needed when using them. However, these solvents have limited clinical application due to their toxicity above certain concentrations. A standard alternative to this drawback is the use of cyclodextrins (CD), oligomers composed of linked glucose monomers (glucopyranose).

The cyclic structure of CD consists of (α-1,4)-linked α-d-glucopyranose units and contains a lipophilic central cavity with the ability of housing hydrophobic molecules and forming inclusion complexes (the structural representation of SBE-β-CD is shown in Supplementary Material S1, Figure S2).

Recent studies have shown the benefits of the complexation of cyclodextrins and isoprenoids, which significantly improve the physicochemical, pharmacodynamic and pharmacokinetic properties of drugs. For example, the use of a complex produced by hosting Aphidicolin (APH) in β-CD was analysed as a cancer treatment [32]. Cabral Silva et al. [33] use a β-CD and FOH inclusion complex to improve the physicochemical and pharmacological properties of FOH. However, the authors only determined which complexation method was more effective to reduce orofacial pain using an animal model, and the effects of the complex on cancer cells was not studied. Moreover, natural cyclodextrins, particularly β-cyclodextrin, have low aqueous solubility [34], limiting their applications. Therefore, to assess the potential application of FOH in anticancer treatments, Sulfobutylated β-cyclodextrin Sodium Salt (SBE-β-CD) was selected as a cyclodextrin because of its high solubility. In addition, SBE-β-CD has the approval of the Food and Drug Administration (FDA), facilitating potential future commercialization. 

SBE-β-CD is a polyanionic cyclodextrin derived from β-CD that is functionalized with a sodium sulfonate salt separated from the lipophilic cavity by a butyl ether spacer group. This cyclodextrin is commercialized as captisol. Due to its structural advantages, it has higher interaction and solubility levels than other cyclodextrins. Furthermore, numerous preclinical and clinical studies assure SBE-β-CD is safe when administered parenterally or orally if used in safe concentration ranges already well-described in the literature, and it does not have the nephrotoxicity associated with traditional beta-cyclodextrins [32,34].

According to the previously mentioned facts, the main objective of this paper is the design and validation of a CD-FOH complex that acts on CDP-choline, reducing membrane formation selectively in cancer cells and, consequently, contributing to the suppression of tumour proliferation.

## 2. Materials and Methods

### 2.1. Reagents

Farnesol 95% (FOH), *E*,*E*-Farnesol 96% (*E*,*E*-FOH), deuterium oxide (D_2_O), Thiazolyl Blue Tetrazolium Bromide, l-α-phosphatidylcholine 99% and Ribonuclease A were purchased from Sigma-Aldrich (Barcelona, Spain) and Sulfobutylated β-cyclodextrin Sodium Salt (SBE-βCD) was purchased on Cyclolab (CY-2041.2).

Phosphate Buffer Saline solution (pH 7.5) was prepared using 136.9 mM Sodium chloride (Sigma), 2068 mM Potassium chloride (Sigma), 10 mM Disodium hydrogen phosphate (Sigma) and 1 mM Potassium dihydrogen phosphate (Sigma). DNA labelling solution was obtained by Cytognos (CYT-PIR-25).

### 2.2. Inclusion Complex Formation

The inclusion complex consisted of a 1:1 molar proportion mixture of FOH and SBE-β-CD at a final concentration of 100 mM, using two types of solvents: D_2_O for further characterization by nuclear magnetic resonance (NMR); and phosphate-buffered saline (PBS) to carry out in vitro tests.

#### 2.2.1. Optimal Time for Complex Formation

The slurry method was followed to study the time required for complex formation [33]. CD and FOH were dissolved in 5 mL of PBS, both at a concentration of 100 mM, continuously stirring at room temperature for 48 h. Samples were taken from the medium at different times, frozen at −20 °C in a conventional freezer and lyophilized with a LyoQuest −55 freeze dryer (Telstar, Tarragona, Spain). The lyophilized powder was suspended in D_2_O to characterize the degree of complexation through NMR (as described in Section 2.2.4).

#### 2.2.2. Temperature Effect on Complex Formation

The influence of temperature on complex formation was studied by dissolving CD and FOH in 0.5 mL of D_2_O, at a concentration of 100 mM for both components. The effect of temperature was analysed by modifying the experimental conditions in the NMR spectrometer, from 25 to 65 °C with an interval of 10 °C.

#### 2.2.3. Stability Studies

Once the complex had been formed, the stability of the complex had to be determined. To this end, the tube containing the complex was kept at room temperature for one month, being periodically checked by NMR to quantify the release of FOH from the complex.

#### 2.2.4. NMR

The NMR spectra were recorded using two different devices: a Bruker WEP-200-SY spectrophotometer at 200 MHz for 1H from the Organic Chemistry Department of the University of Salamanca and a Bruker Avance Neo 400 MHz DRX with BBO cryoprobe spectrophotometer at 400 MHz for 1H and 100 MHz for 13C from the Nuclear Magnetic Resonance Service of the University of Salamanca.

The proton chemical shifts (δ) were reported in parts per million (ppm), and the signals relative to the solvent residual peak as an internal standard of the solvent (D_2_O—4.79 ppm) and tetramethylsilane (TMS 0.0 ppm). Coupling constants (J) have been expressed in hertz (Hz) and the following abbreviations have been used to explain multiplicities: s (singlet), d (doublet), t (triplet), c (quartet), q (quintuplet), dd (double doublet), dt (double triplet), and m (multiplet).

Spectra were analysed with MestReNova v6.0.5475 (Mestrelab Research S.L.). The FOH/SBE-β-CD molar ratios were calculated by direct NMR integration of their appropriate signals. The concentration of SBE-β-CD was constant at 0.1 mM and FOH concentration was calculated according to Equation (1):

(1) $${\left[\;\right]}_{\mathrm{FOH}}=\frac{\frac{\delta_{\mathrm{FOH}}}{{\mathrm{MW}}_{H}\cdot {\;n}_{H}}}{\frac{\delta_{\mathrm{SBE}\;-\;\beta \;-\;\mathrm{CD}}}{{\mathrm{MW}}_{H}\cdot {\;n}_{H}}}\cdot {\left[\;\right]}_{\mathrm{SBE}\;-\;\beta \;-\;\mathrm{CD}}$$

#### 2.2.5. UV Spectrophotometry

Spectrophotometry was carried out to corroborate the NMR results. Absorbance spectrums were performed on a Shimadzu UV-1800 spectrophotometer. The scan was performed for 3 repetitions at a fast measurement speed in a range of 900–190 nm with a pitch of 2 nm. Finally, they were analysed with UVProbe Software (2.42, Shimadzu Scientific Instruments, Tokyo, Japan).

### 2.3. Validation of Inclusion Complex by In Vitro Tests

#### 2.3.1. Cell Lines and Culture Conditions

To analyse the effects of the CD-FOH complex, four cell lines were used for in vitro studies (three tumour cells and a normal one): fibroblast normal HS5 (ATCC^®^ CRL11882™), colorectal carcinoma HCT-116 (ATCC^®^ CCL247™), hepatocellular carcinoma HepG2 (ATCC^®^ HB8065™), breast carcinoma BT-474 (ATCC^®^ HTB20™). The cell lines were obtained from the American Tissue Collection (Manassas, VA, USA).

All cells were cultured in Dulbecco’s Modified Eagle Medium supplemented with 10% fetal bovine serum (DMEM+) (Gibco) and 1% penicillin-streptomycin (Corning). They were maintained at 37 °C in a 5% CO_2_ atmosphere. At 70–90% confluence, the cells were sub-cultured using 0.05% trypsin/EDTA 1× (Gibco).

#### 2.3.2. In Vitro Tests

To evaluate an anticancer agent, it is essential to determine its cytotoxicity with in vitro tests. Farnesol toxicity was evaluated in its free form (FOH in DMSO) and in the complex form (with SBE-β-CD in PBS). 

The first step involved the calculation of the half maximal inhibitory concentration (IC50) of each treatment. Stock solutions of FOH at 50 mM were prepared and added to DMEM+, reaching final FOH concentrations between 0 and 750 µM, which implies a final DMSO concentration between 0 and 1.5% (vol). Cell viability was calculated as indicated below (Section 2.3.3). In order to perform a comparative trial of the treatments, the optimal dose was chosen following the same procedure as for the IC50 test.

Given the price difference in FOH isomers, an analysis was carried out to observe if they presented any variation in cytotoxicity levels. Results of the mix of isomers were compared (under the optimal conditions determined in Section 2.2) with the use of single *E*,*E*-FOH (in free or complex form) to determine if the racemic mixture presented any difference in the therapeutic effect of FOH. 

Finally, as mentioned in the introduction, one of the FOH mechanisms of action involves the inhibition of the CDP-choline enzyme. This inhibition reduces PC synthesis, decreasing cell membrane formation and, therefore, cell proliferation. Since normal cells have a slower growth rate and a correspondingly reduced need to create new membrane components, FOH is expected to affect them less [3]. To verify this statement, another in vitro culture was carried out by supplementing the cell medium using PC and monitoring the cell growth in the presence of FOH (free or complex) at the same concentration of PC.

#### 2.3.3. Cell Viability

An MTT assay was performed in cells in exponential growth to measure cell viability. The cells were plated by quadruplicate in flat-bottomed 24-well plates (Falcon) at an initial concentration of 16,000 cells per well. Plates were incubated for 24 h in a 5% CO_2_ humidified incubator, and cell viability was tested by incubating cells with different treatments dissolved in DMEM+. In all tests, plates included medium control and cell control.

The MTT assay is a colorimetric assay to determine the cytotoxicity of certain drugs (measuring cell viability). Thiazolyl Blue Tetrazolium Bromide dissolved in PBS at 5 mg/mL was added to the cells (20 μL/well), reacting with living cells and producing violet formazan crystals. After incubation at 37 °C for 1 h, the DMEM+ medium was removed, and the crystals were dissolved by adding 0.5 mL DMSO. Absorbance was measured using ultraviolet light (λ = 550 nm) by means of an EZ Read 2000 Microplate Reader (Biochrom). Cell viability was calculated as indicated in Equation (2) [30].

(2) $$\mathrm{cell}\;\mathrm{viability}\;\left(\%\right)=\frac{\mathrm{average}\;\mathrm{OD}\;\mathrm{wells}-\mathrm{average}\;\mathrm{OD}\;\mathrm{wells}\;\mathrm{medium}\;\mathrm{control}}{\mathrm{average}\;\mathrm{OD}\;\mathrm{control}\;\mathrm{wells}-\mathrm{average}\;\mathrm{OD}\;\mathrm{wells}\;\mathrm{medium}\;\mathrm{control}}$$

#### 2.3.4. Cell Cycle Analysis

Flow cytometry analysis was carried out in order to corroborate the hypothesis sustaining that FOH acts as an inducer of cell cycle arrest, causing apoptosis [3,12,23].

Fibroblast (HS-5) and colorectal cancer (HCT-116) cells were seeded onto 6-well plates at a density of 2 × 105 cells/well and stabilized by incubating them for 24 h at 37 °C. After treatment using FOH for 24 h and 48 h, the cells were collected and washed with 1× PBS. Cell pellets were resuspended in 1 mL of DNA labelling solution, which contained propidium iodine (CYT-PIR-25, Cytognos, Santa Marta de Tormes, Spain), and stained for 30 min at room temperature in the dark. The DNA contents of the stained cells were analysed using Cell Quest Software with a FACScan Calibur flow cytometry (Becton-Dickinson, Franklin Lakes, NJ, USA).

In this experiment, 6-well plates were used instead of the 24-well ones, therefore needing a higher cell concentration than in the viability tests. Two different FOH concentrations were tested since the optimal dose had to reduce cell viability but not cause total apoptosis (150 µM for free FOH and 300 µM for FOH in its complex form with cyclodextrin).

### 2.4. Statistical Analysis and Parameters Estimation

The data analysis was performed using Statgraphics Centurion XVI Version 16.1.03 (StatPoint Technologies, Inc. 1982–2010, Warrenton, VA, USA). This procedure performed a one-way analysis of variance (ANOVA) for absorbance at 550 nm (MTT assay). The F-test in the ANOVA table determined if there were significant differences between the means. Differences were considered significant with a confidence interval of 95% (*p* < 0.05).

The data from the previously described IC50 tests were used to model the response of cell lines to FOH treatment. IC50 was calculated by parameter estimation (Hessian) of Equation (3), which expresses the variation of cell viability in percentage (y) according to the dose administered (D):

(3) $$\frac{\mathrm{dy}}{\mathrm{dD}}=\frac{-100\cdot n\cdot {\left(\frac{D}{{\mathrm{IC}}_{50}}\right)}^{n}}{D{\left[{\left(\frac{D}{{\mathrm{IC}}_{50}}\right)}^{n}+1\right]}^{2}}$$

where n is the order of the inhibition and the IC50 expresses the dose at which the inhibition process reaches 50% of the cells. All the simulations were carried out by considering the cell viability results at 48 h.

The equation was implemented in gPROMS 6.0 (PSE) and a Hessian estimation was carried out by parameter estimation (n and IC50 are estimated). The constant variance model was selected and the sensor for parameter estimation was fixed as a function of the viability variable (y) in a range between 0.005 and 0.10.

Boundary conditions were set for each parameter: IC50 could take values from 10 µM to 2000 µM and n could take values from 0.1 to 10. For statistical analysis, the goodness of fit was performed by comparing the value of weighted residuals (WR) with the chi-square value (X2) and a confidence interval of 95%. On the other hand, the lack of fit test was performed by comparing the F-value with F-critical. The accuracy of a model is generally considered precise if the value of WR is less than X2 and the F-value is lower than F-critical.

Parameter estimation was performed in a personal computer Intel Core I3 3.70 GHz and 4 GB RAM.

## 3. Results and Discussion

The results section begins with a description of complex formation, followed by the results of in vitro tests. Firstly, the dose-response results are shown, both for the free form drug and the complex. Secondly, once the IC50 had been established, a comparative test of the free form drug and the complex at the same concentration is described. Finally, the last two assays compare pure FOH and a mixture of isomers, as well as how the addition of PC would reverse the action of FOH.

### 3.1. Complex Formation (SBE-β-CD-FOH and SBE-β-CD-E,E-FOH)

**Figure 2:**
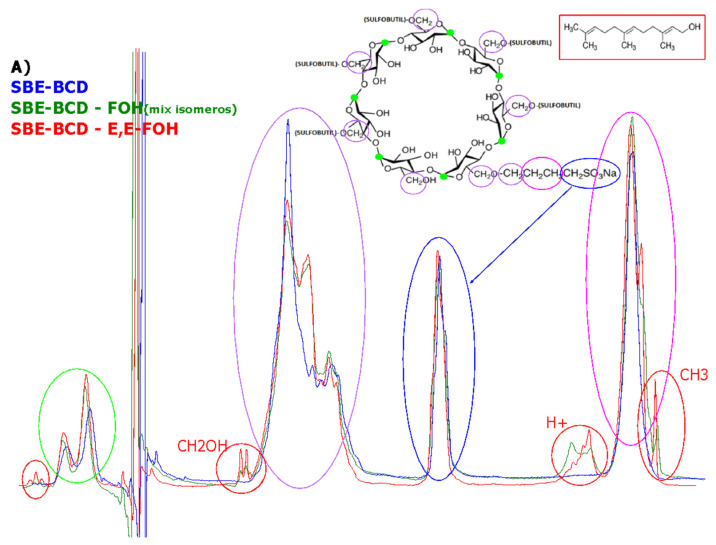

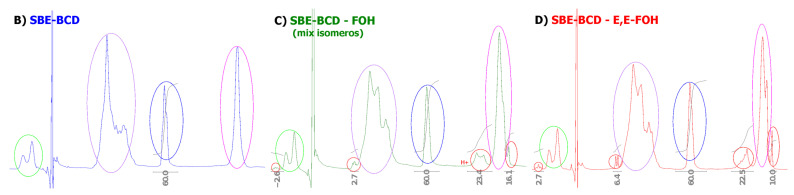
NMR Spectra of SBE-β-CD (**B**), complex with FOH isomer mixture (**C**), complex *E*,*E*-FOH (**D**), and its comparative (**A**).

Firstly, the complex formation was studied following the methodology described in Section 2.2. The amount of FOH included in SBE-β-CD cyclodextrin was determined by NMR, comparing the complex spectrum and SBE-β-CD spectrum (Figure 2). One single peak was identified for FOH at 4.35–4.75 ppm. As this peak did not overlap with the signal of SBE-β-CD, it could be used for quantifying the yield of complexation. The established CD concentration for this experiment (100 mM) presented another single peak at 5.4 (without interferences).

**Figure 3:**
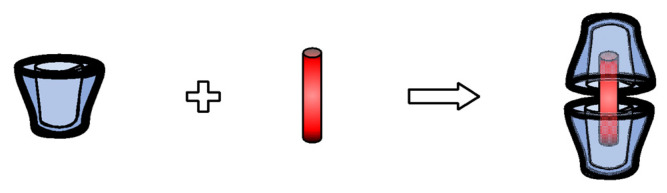
Formation cyclodextrin—drug inclusion complexes [35].

By comparing the integral value for both peaks, it was possible to estimate the amount of FOH included in the well (the calculation of proportion ratio FOH-CD is shown in Supplementary Material Equation (S3)). The results indicated that there were 2 moles of CD for each mole of FOH. Based on this numerical result, a conformation of the inclusion complex could be proposed. Figure 3 indicates a possible structure of the inclusion complex based on the results of stoichiometry (2:1).

In terms of complex stability, no significant differences were found in the spectra of the samples collected at different times for one month. Moreover, temperature does not affect the inclusion rate and the complexation process can be considered instantaneous. (The series of NMR spectra are presented in Supplementary Material S1 Figures S4 and S5).

The spectrum of the sample can be compared with the spectra of serial dilutions of the different types of FOH dissolved in ethanol (Supplementary Material S1, Figure S6). In this way, the amount of FOH obtained is consistent with the results from the NMR.

Size and zeta potential were analysed by DLS (Supplementary Material S1, Figure S7); it is possible to observe that results offer a particle size around 1–2 nm, which is the size reported in literature for cyclodextrins; however, this is the detection limit of DLS technique, and therefore the results are not conclusive.

### 3.2. Therapeutic Effect (In Vitro Tests)

#### 3.2.1. Dose-Response Modelling

**Figure 4:**
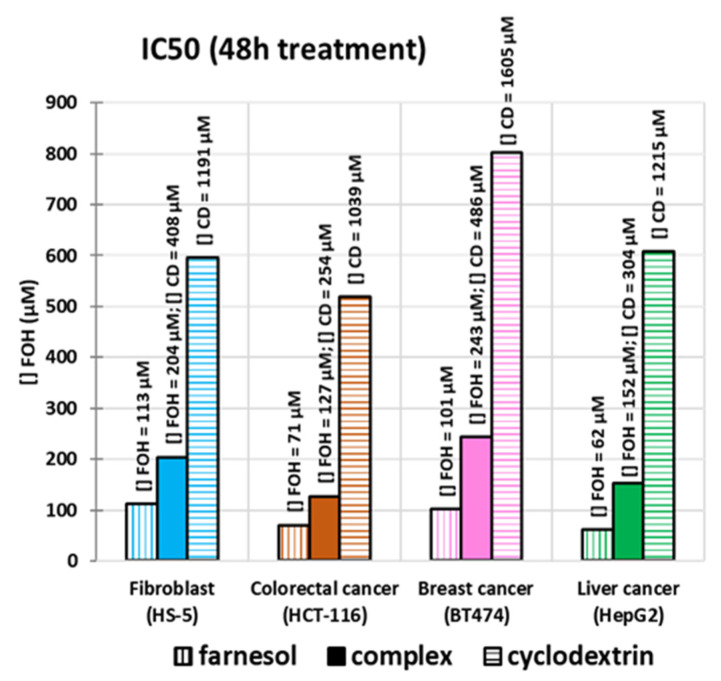
Graphical representation of IC50 estimation for FOH (vertically hatched columns), CD (horizontally hatched columns), and the complex (filled columns) in four cancer cell lines (blue—fibroblast, orange—colorectal, pink—breast, green—liver).

According to the Materials and Method sections, parameters of the dose-response model were predicted by Hessian estimation using gPROMS software. Table 1 showed the results of the parameters estimated (IC50 and n) for the four cell lines studied (fibroblasts, colorectal, breast, and liver). Moreover, the table presents the values of statistical analysis (variance value, goodness of fit and lack of fit test) as well as some data related to execution (the number of non-linear iterations and the CPU time required). Likewise, a graphical representation of IC50 is presented in Figure 4.

According to Table 1, IC50 for CD is considerably higher than the one for FOH and the complex. Therefore, the toxicity of CD can be considered negligible for the doses to be used. In general, the inclusion of FOH in CD doubled the IC50. There is a significant difference between two cancer cell lines (liver and colorectal) and normal cell lines (fibroblasts). This difference can be an advantage for treatment, as it implies that the inclusion is selective towards cancer cells without damaging normal ones.

All the estimations have passed both tests (goodness of fit and lack of fit), with an average of 24 iterations. Most of them had a CPU time of less than 1 s, and a variance average of 4.9% (with only one simulation on the 10% limit).

The curve fitting graphs are presented in the Supplementary Material S2 (S8). The results from IC50 were used for the design of the next experiments to validate the therapeutic effect.

#### 3.2.2. FOH Effects on Cell Viability

**Figure 5:**
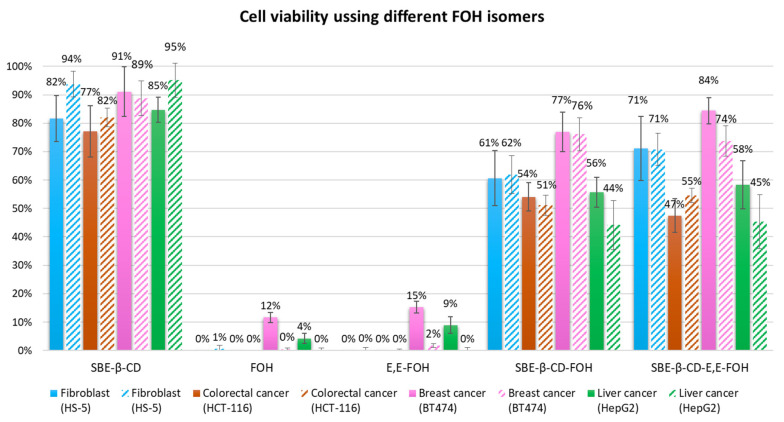
Cell viability results of each cell line treated for 24 h (filled columns) and 48 h (hatched columns).

Based on the results predicted by the Hessian estimation previous described, a comparative test of the treatments (free FOH and SBE-β-CD—FOH complex) was carried out with a final FOH concentration of 150 μM. Figure 5 shows cell viability results for each of the cell lines treated using CD and FOH separately, and in the complex form after comparing the average absorbance with their respective control at each time.

The difference between absorbances is better appreciated after 48 h, making the results more reliable (Figure 5). At this time, CD control did not show a significant decrease in viability, except in the case of the liver line, in which it only reduced up to 80%. The complexes showed less reduction in cell viability than the free form. Viability of colon and liver cells remained at 50%, while free FOH reduced it up to 31–36% respectively. In the case of the breast line, there was a smaller difference between the free form and the complex, where the viability was 85% and 74%, respectively. The best results were obtained by the normal cell line, since free FOH reduced its viability up to 61%, and the complex had a lower inhibition, keeping it above 80%. 

#### 3.2.3. FOH Isomers Effect on Cell Viability

As it was mentioned in the introduction, the previous studies in literature have only used one type of FOH isomer (*E*,*E*-FOH). However, due to its high price, it was necessary to study if a mix of isomers offered the same result as the purified isomer (*E*,*E*-FOH). According to Figure 5, there are no significant differences between the different types of FOH (neither the free form nor the complex). Hence, it is not necessary to use *E*,*E*-FOH. This would reduce the cost of a potential treatment with this isoprenoid, as its price is 10% of the one for trans-trans isomer.

#### 3.2.4. PC Effect of FOH on Cell Viability

**Figure 6:**
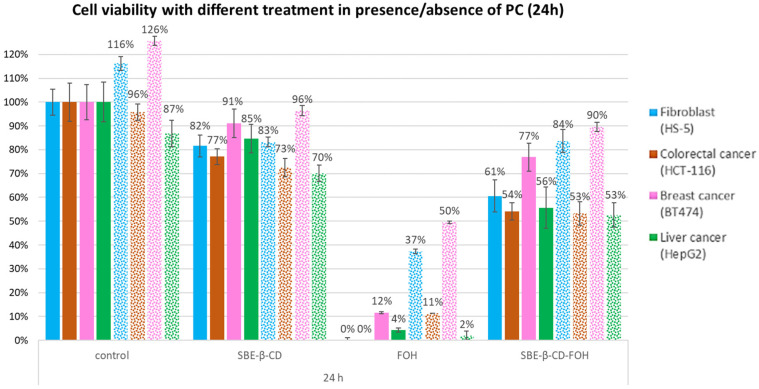
Cell viability results for each cell line treated for 24 h in absence (filled columns) or presence (dotted columns) of PC.

Finally, in order to corroborate the mechanism used by FOH to reduce cell proliferation (by inhibiting the CDP-choline enzyme), another in vitro culture test was carried out. For this test, 150 μM of phosphatidylcholine (PC) was added to the cell medium following the protocol described in Section 2.3.3. Figure 6 shows the results of this experiment, indicating that controls were not significantly altered by the presence of PC, except in the case of the breast line, where an increased proliferation was observed. Cell viability in cells treated using FOH is increased in the presence of PC at 24 h, but this effect disappears at 48 h (it is shown in Supplementary Material S3, Figures S9 and S10). 

A possible explanation for this phenomenon could be that cells may have consumed all the available PC in the medium. These results indicate that the inhibition of CPD-choline is one of the main FOH pathways of action, having a remarkable effect on tumour cell proliferation. In addition, the amount of PC available in the medium would control the reversibility of the treatment.

### 3.3. Cell Cycle Analysis

**Figure 7:**
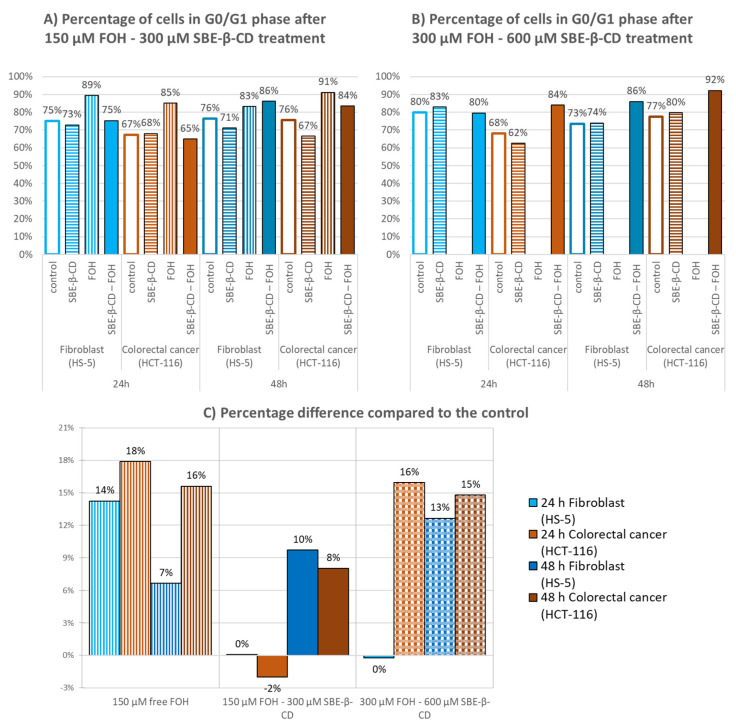
Percentage of cells in G0/G1 phase after treatment of fibroblast and colorectal cancer cells for 24 and 48 h; (**A**) Concentration of treatments is FOH 150 µM and SBE-β-CD 300 µM; (**B**) Concentration of treatments is FOH 300 µM and SBE-β-CD 600 µM. (**C**) Difference in the percentage of cells in G0/G1 phase compared to the control.

To determine the effect FOH has on the cell cycle, propidium iodide-stained cells were analysed using flow cytometry as described in Section 2.3.4. Figure 7A shows the results of cytometry for HS5 and HCT116 cells treated with 150 μM FOH and Figure 7B shows the effect in the same lines with double FOH concentration (300 μM). The use of these two different concentrations allowed us to identify the effects of the complex and the free form drug in cell cycle arrest. 

In both cases, CD at this concentration did not affect cell cycle arrest, whereas FOH caused a substantial increase in the G1/G0 population due to the activation of cell cycle arrest mechanisms. For a better understanding, the percentage differences were calculated and summarized in Figure 7C.

The original graphics was obtained using Infinicyt programme, and they are shown in Supplementary Material S5, Figure S11.

Free FOH at 300 µM destroyed all cells, and FOH at 150 µM increased the percentage of cells in G0/G1 phase in both cell lines after 24 h, although it was higher in the case of colorectal cancer cells. The complex with an FOH concentration at 150 µM showed no effects, with just a slight increase after 48 h. However, when using FOH at 300 µM, the complex increased the percentage of colon cancer cells by 16% after 24 h. After 48 h, this percentage increase was maintained, while normal cells increased by 23%.

## 4. Conclusions

According to the tests performed, temperature had no effects on the complexation process or complexation kinetics. The obtained results presented no difference, and the complexation process was instantaneous, obtaining a solution containing 1 mole of FOH for every 2 moles of CD.

Regarding cell viability assays, FOH was selective, and it affected the viability of colon and liver cancer cells more than breast cancer cells and fibroblasts. The IC50 values obtained for free FOH were 71, 62, 101, and 113 μM, while the concentrations obtained for the inclusion complex were 127, 152, 243, and 204 μM. When the free form of FOH at 150 μM was used, the cell viability was reduced up to 0%. However, cell viability was reduced when the cells were treated using the complex form, reaching 50% in the case of colon and liver, 60% in fibroblasts, and only 75% in breast cancer.

In addition, both types of isomers obtained the same results, which indicates a potential reduction in the cost of future cancer treatments.

## Figures and Tables

**Table 1:** Parameter estimation for fitting the data to dose-response curve.

Cell Line	Treatment	IC50 (uM)	n	Weighted Residuals	X2	F-Value	F-Critical	NLP Iterations	CPU Time (s)	Variance (%)
LIVER (HepG2)	CD	1215	1.3	7	9.5	0.8	4.1	23	0.9	3.20
	FOH-CD	152	2.7	7	9.5	0.8	4.1	52	2.8	2.80
	FOH	62	2.9	7	9.5	0.8	4.1	13	0.4	4.10
BREAST (BT-474)	CD	1605	1.6	7.1	9.5	0.8	4.1	19	0.6	5.50
	FOH-CD	243	4.2	7	9.5	0.8	4.1	23	1.0	2.00
	FOH	101	6	7	11.1	0.4	4	12	0.4	10.0
COLORECTAL (HCT-116)	CD	1039	0.9	9	12.6	0.5	3.4	31	1.1	3.70
	FOH-CD	127	1.9	9	12.6	0.8	4.1	14	0.4	5.60
	FOH	71	2.7	9	12.6	0.5	3.4	38	1.5	6.40
FIBROBLAST (HS-5)	CD	1191	1	9	12.6	0.5	3.4	28	0.6	2.70
	FOH-CD	204	3.2	7	9.5	0.8	4.1	16	0.7	4.00
	FOH	113	6.7	10	14.1	0.4	3.1	15	0.4	8.60

n: parameter from IC50 model, X2: chi-squared value, NLP: nonlinear programming.

## Data Availability

Not applicable.

## Supplementary Materials

The following supporting information can be downloaded at: https://www.mdpi.com/article/10.3390/molecules27092735/s1, Figure S1: Chemical structure of FOH isomers; Figure S2: Cyclodextrin structure; Figure S3: Calculation of proportion ration FOH-CD; Figure S4: Series of NMR spectra (stability); Figure S5: Series of NMR spectra at different temperatures; Figure S6: UV-Vis spectrums of dilutions of different types of FOH; Figure S7: DLS and Z-potential; Figure S8: Curve fitting for IC50 estimation; Figure S9: Effect of external addition of PC; Figure S10: variation of cell viability (48 h) in presence of PC; Figure S11: ANOVA tables; Figure S12: cell phase results.

## Sample Availability

Samples of the compounds SBE-β-CD, *E*,*E*-FOH, mix isomers of FOH and the complex are available from the authors.
