# Supplementary material for: An Approach to Minimize Tumour Proliferation by Reducing the Formation of Components for Cell Membrane
Source: Molecules. 2022 Apr 24;27(9):2735. doi: 10.3390/molecules27092735 (PMC9105759; doi:10.3390/molecules27092735)
Supplement: Supplementary file 1 [file molecules-27-02735-s001.zip › molecules-1662931-supplementary.pdf]

# An approach to minimize tumour proliferation by reducing the formation of components for cell membrane

Inmaculada de Dios-Pérez, Álvaro González-Garcinuño

and Eva María Martín del Valle*

# Supplementary Material

## Supplementary material S1

## Chemical characterization of FOH isomers and cyclodextrin structure

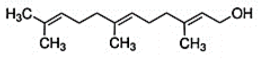


: trans, trans – farnesol (CAS 106-28-5)

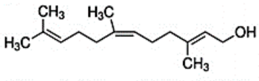


:  2-trans, 6-cis – farnesol (CAS 3879-60-5)

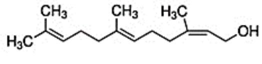


: 2-cis, 6-trans – farnesol (CAS 3790-71-4)

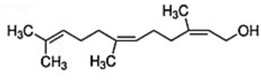


: cis, cis – farnesol (CAS 16106-95-9)

*Figure S1. Structural representation of the different geometric isomers of farnesol.*

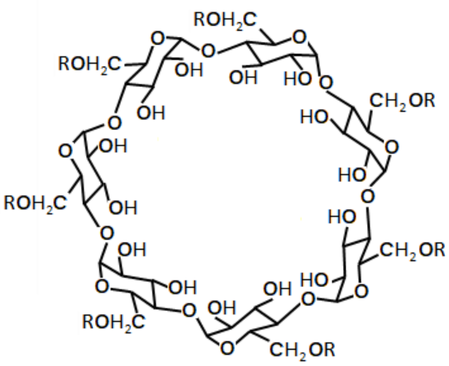

R = -H or sulfobutyl (-$CH_2)_4$-$SO_3Na$)
Empirical Formula: $C_{42}H_{70-6.4}O_{35} \cdot (C_4H_8SO_3Na)_{6.4}$

*Figure S2. Structural representation of SBE-βCD.*

$$[\,]_{FOH} = \frac{\frac{\delta_{FOH}}{MW_{FOH}}}{\frac{\delta_{SBE-\beta-CD}}{MW_{SBE-\beta-CD}}} \cdot [\,]_{SBE-\beta-CD} = \frac{\frac{23{,}4}{10\,H}}{\frac{60{,}0}{6{,}4 \cdot 2\,H}} \cdot 100\,mM = \frac{2{,}34\,mol\,FOH}{4{,}69\,mol\,CD} \cdot 100\,mM \approx 50\,mM$$

$$[\,]_{E,E-FOH} = \frac{\frac{\delta_{E,E-FOH}}{MW_{E,E-FOH}}}{\frac{\delta_{SBE-\beta-CD}}{MW_{SBE-\beta-CD}}} \cdot [\,]_{SBE-\beta-CD} = \frac{\frac{22{,}5}{10\,H}}{\frac{60{,}0}{6{,}4 \cdot 2\,H}} \cdot 100\,mM = \frac{2{,}25\,mol\,FOH}{4{,}69\,mol\,CD} \cdot 100\,mM \approx 50\,mM$$

*Equation S3. Calculation of proportion ratio FOH-CD for isomers mix (first line) and E,E-FOH (second line).*

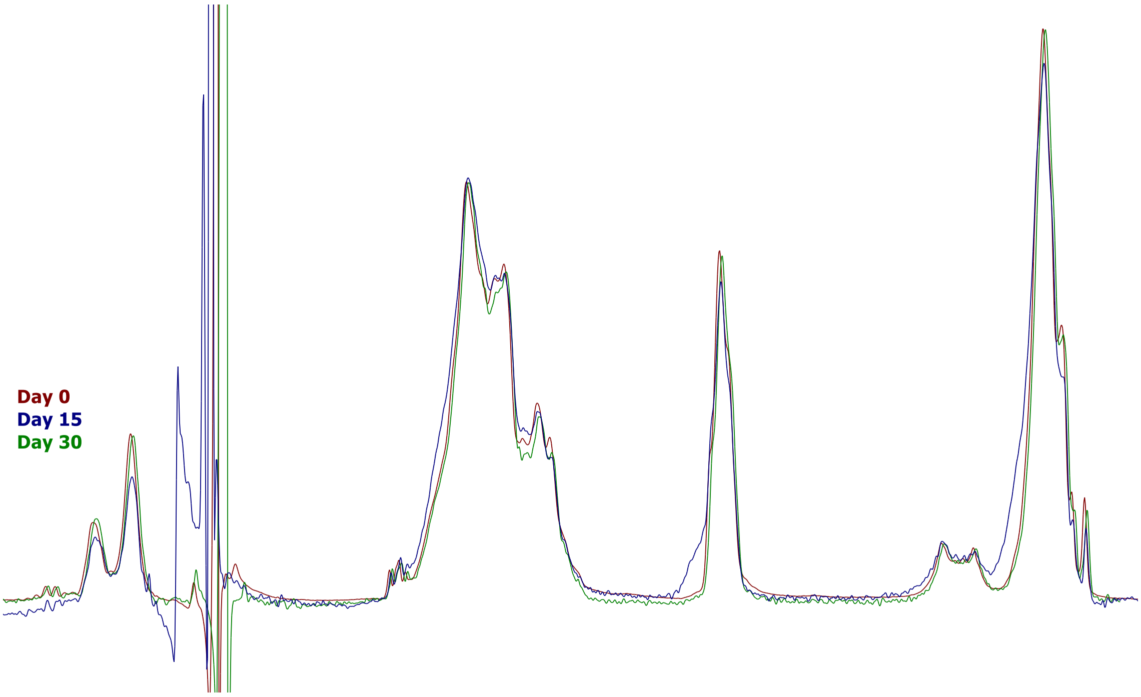


*Figure S4: A: NMR-spectra for the complex CD-FOH of the samples collected at different times for one month.*

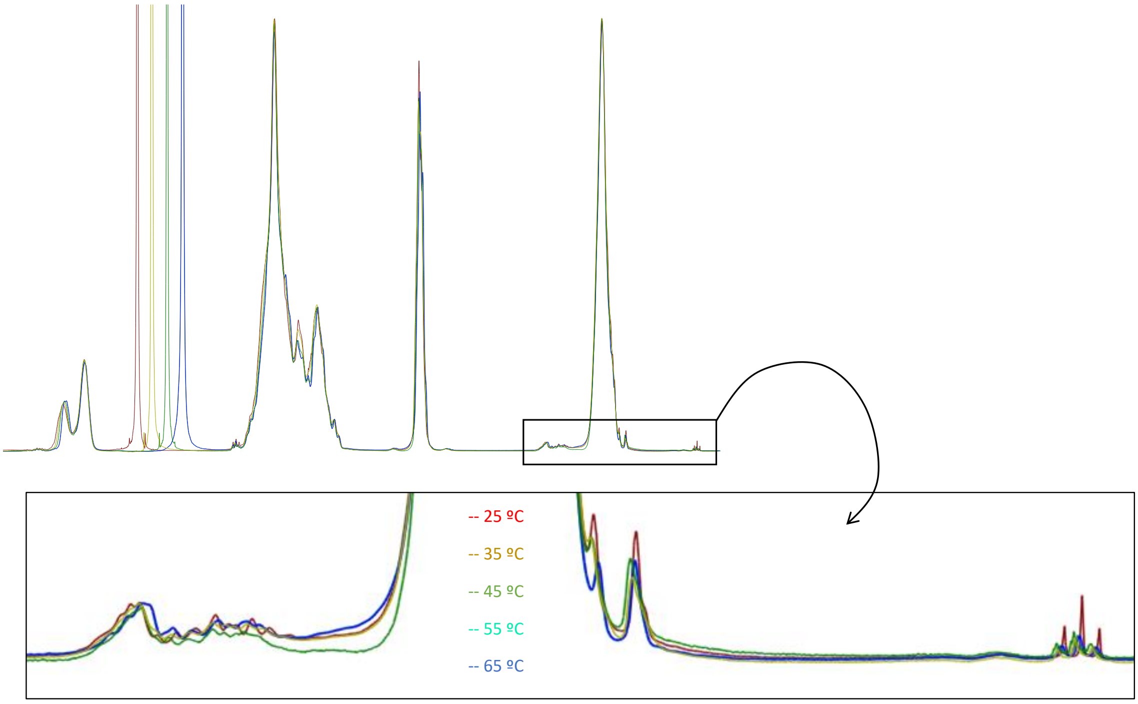


*Figure S5: A: NMR-spectra for the complex CD-FOH working with different temperatures.*

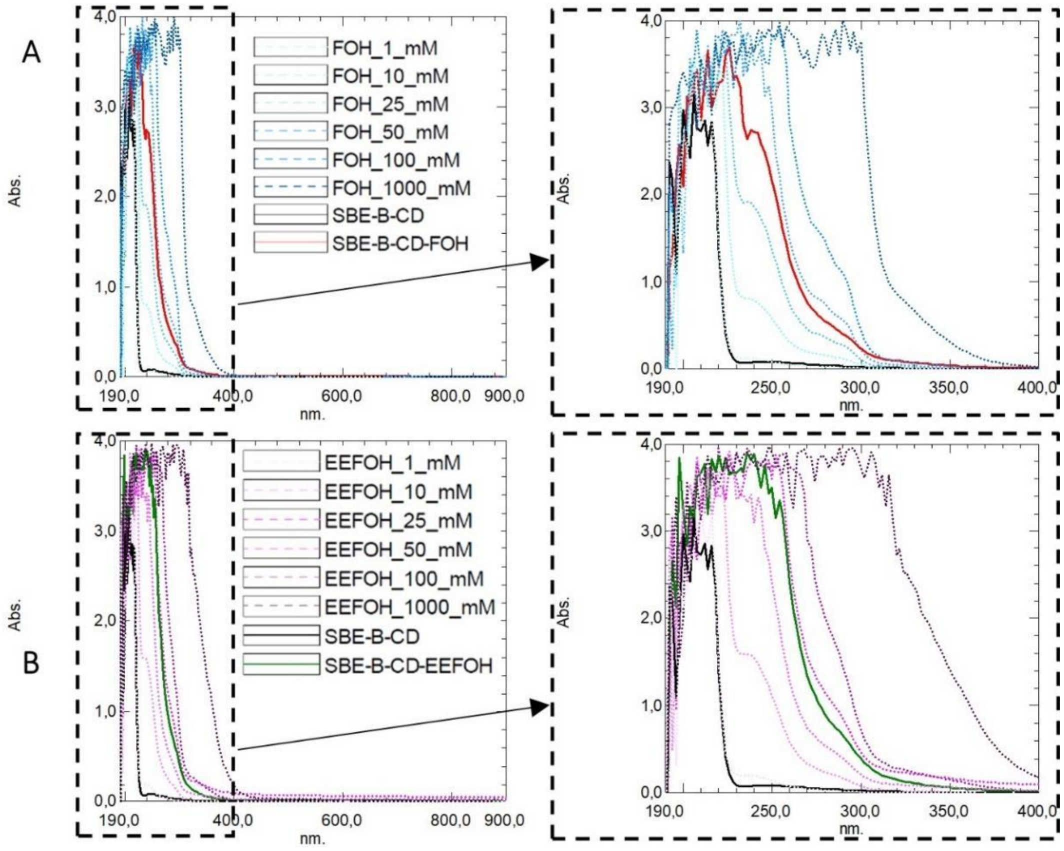


*Figure S6: A) UV-Vis spectra for free FOH at different concentrations in ethanol, SBE-β-CD and inclusion complexes (with FOH). B) UV-spectra for free E,E-FOH, SBE-β-CD and inclusion complexes (with E,E-FOH).*

**A**

**System**

| | | | |
|---|---|---|---|
| **Temperature (°C):** | 25,0 | **Duration Used (s):** | 500 |
| **Count Rate (kcps):** | 6,9 | **Measurement Position (mm):** | 4,65 |
| **Cell Description:** | Disposable sizing cuvette | **Attenuator:** | 11 |

| | | | **Size (d.nm):** | **% Intensity:** | **St Dev (d.nm):** |
|---|---|---|---|---|---|
| **Z-Average (d.nm):** | 1,392 | **Peak 1:** | 1,389 | 91,2 | 0,4730 |
| **PdI:** | 0,169 | **Peak 2:** | 1546 | 8,8 | 954,8 |
| **Intercept:** | 0,806 | **Peak 3:** | 0,000 | 0,0 | 0,000 |
| **Result quality :** | **Refer to quality report** | | | | |

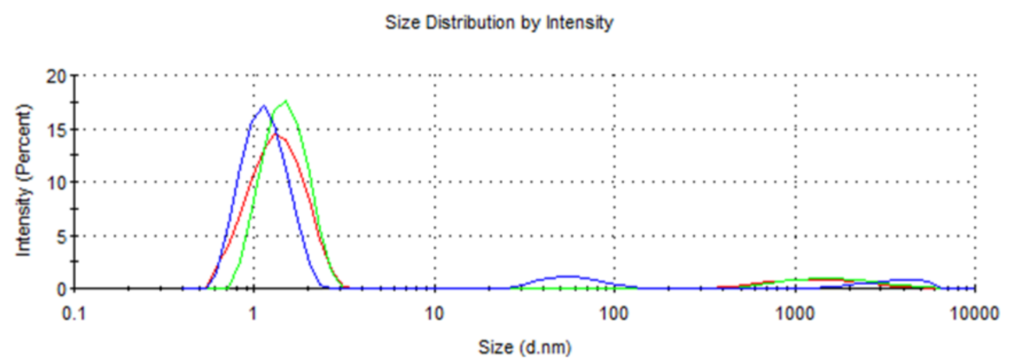


**B**

**System**

| | | | |
|---|---|---|---|
| **Temperature (°C):** | 25,0 | **Zeta Runs:** | 100 |
| **Count Rate (kcps):** | 13,9 | **Measurement Position (mm):** | 2,00 |
| **Cell Description:** | Clear disposable zeta c... | **Attenuator:** | 11 |

**Results**

| | | | **Mean (mV)** | **Area (%)** | **St Dev (mV)** |
|---|---|---|---|---|---|
| **Zeta Potential (mV):** | 0,00329 | **Peak 1:** | 0,00329 | 100,0 | 3,56 |
| **Zeta Deviation (mV):** | 3,56 | **Peak 2:** | 0,00 | 0,0 | 0,00 |
| **Conductivity (mS/cm):** | 3,48e-4 | **Peak 3:** | 0,00 | 0,0 | 0,00 |
| **Result quality** | **See result quality report** | | | | |

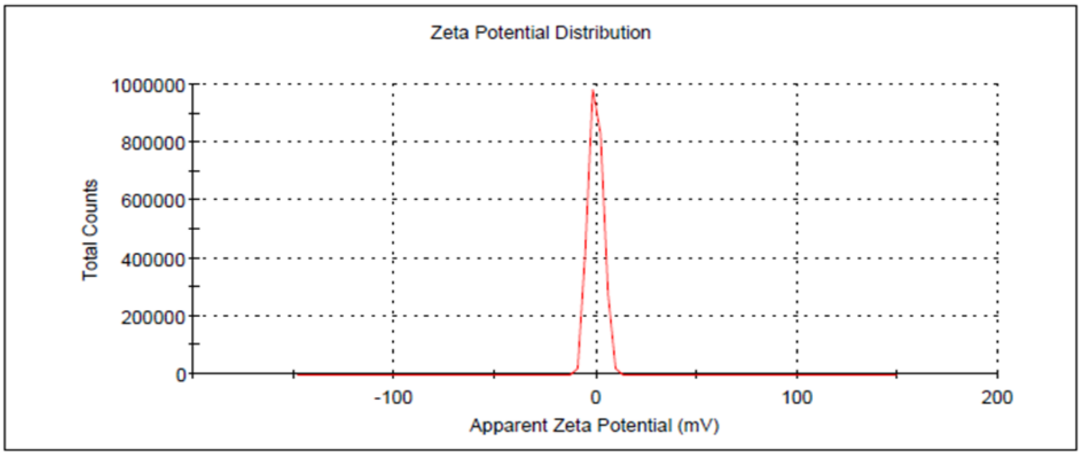


*Figure S7: Results size (A) and zeta potential (B) of SBE-β-CD - FOH by DLS.*

**Supplementary material S2**

**Curves of dose response modelling**

## CYCLODEXTRIN ESTIMATION

### FIBROBLAST NORMAL

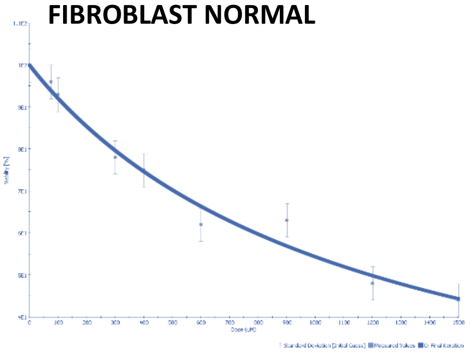

### COLORECTAL CANCER

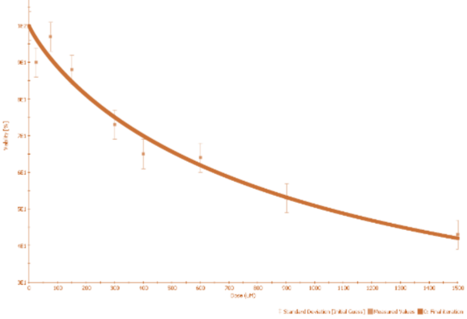

### BREAST CANCER

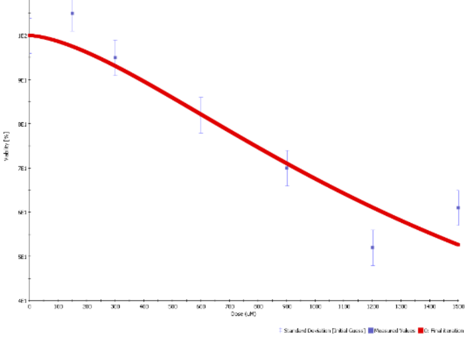

### LIVER CANCER

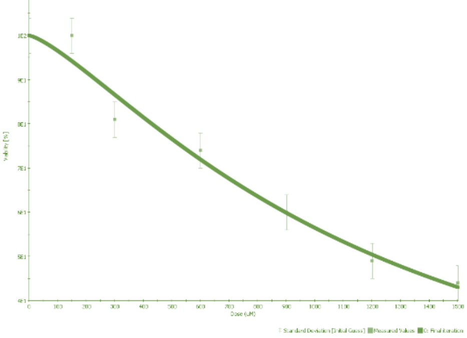

## FARNESOL ESTIMATION

### FIBROBLAST NORMAL

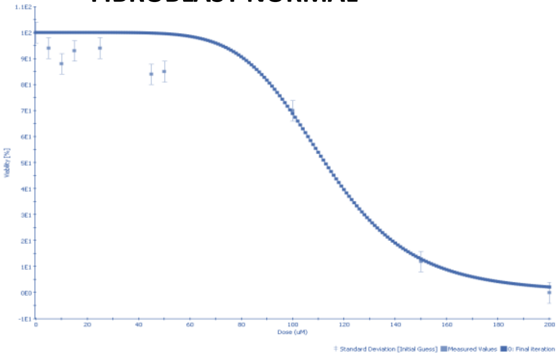

### COLORECTAL CANCER

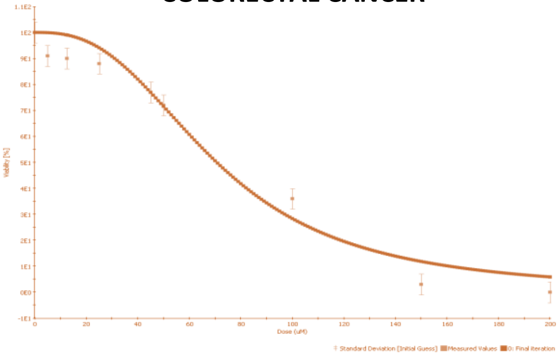

### BREAST CANCER

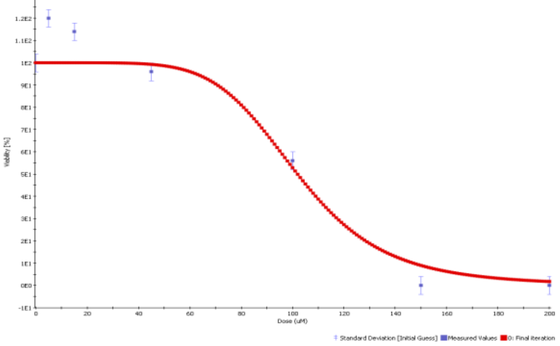

### LIVER CANCER

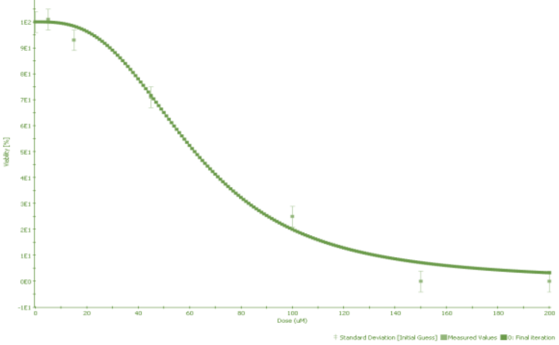

# COMPLEX ESTIMATION

### FIBROBLAST NORMAL

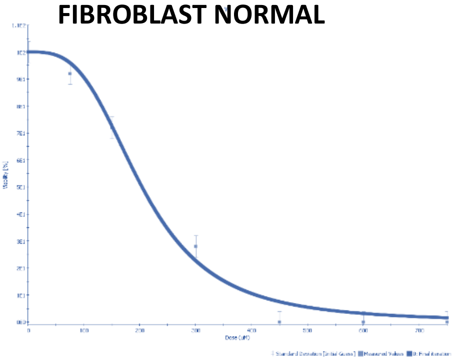

### COLORECTAL CANCER

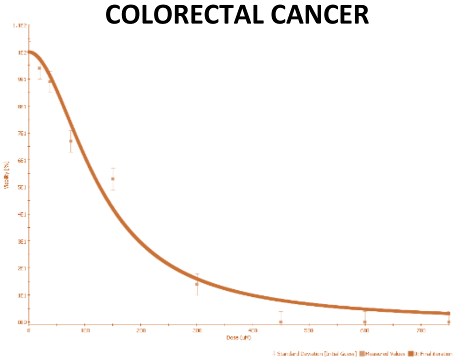

### BREAST CANCER

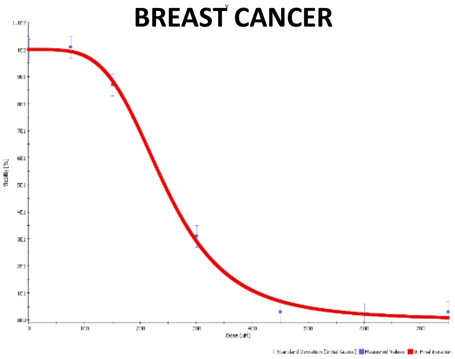

### LIVER CANCER

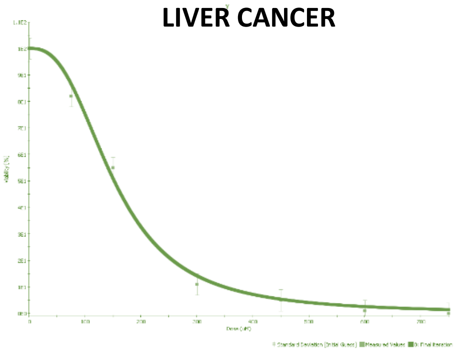

*Figure S8: Curves of dose response modelling.*

## Supplementary material S3

## Addition of external PC: in vitro results

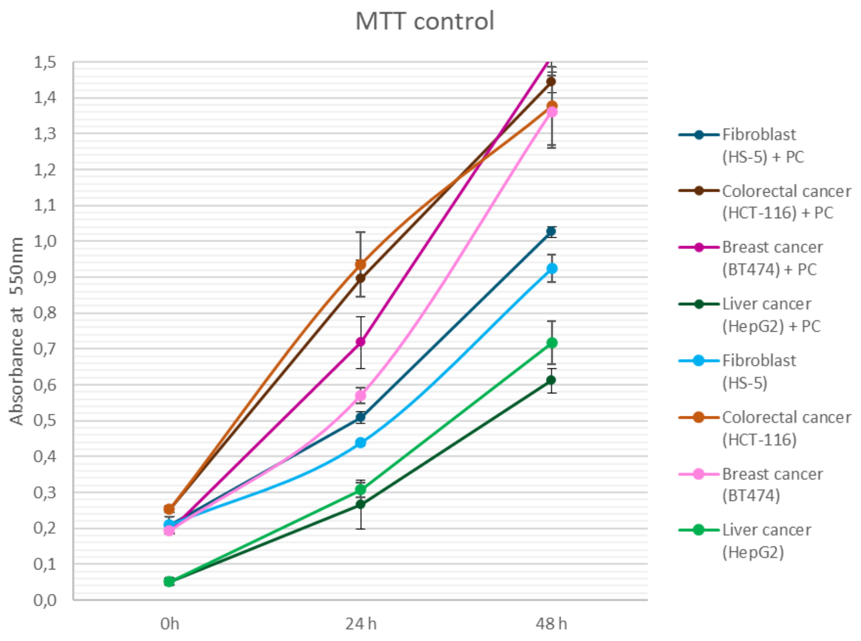


*Figure S9: Effect of external addition of PC. Absorbance raw data for four cell lines (with or without addition of PC).*

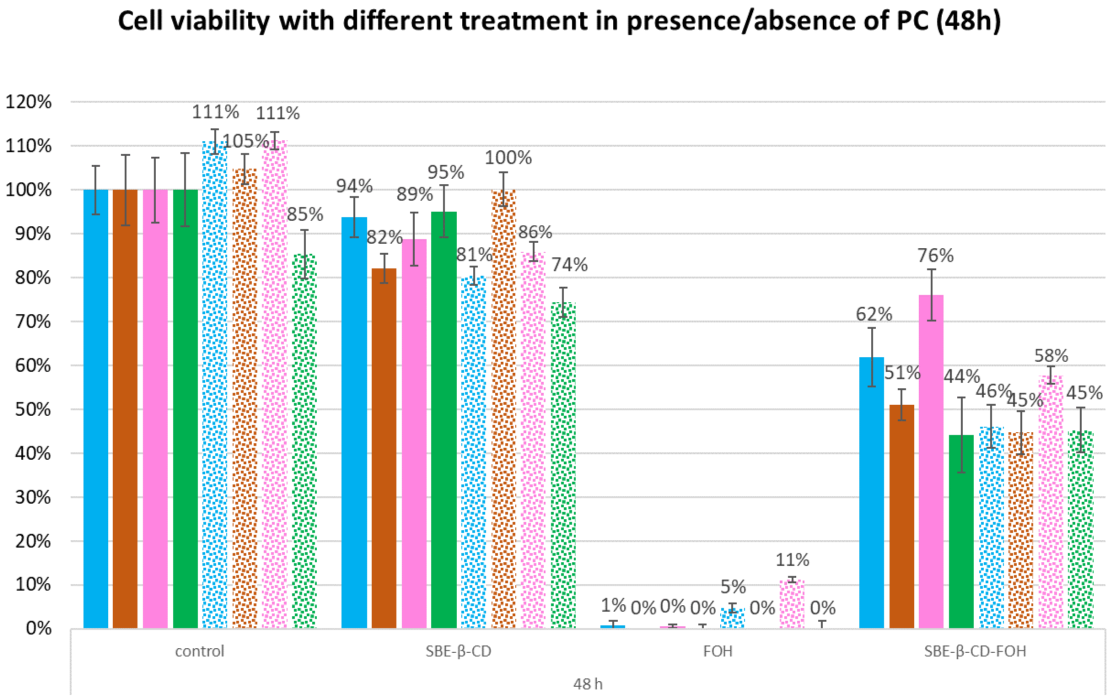


*Figure S10: Effect of external addition of PC. Cell viability results calculated from Figure S8.*

# Supplementary material S4

## Statistical analysis: ANOVA tables

**ANOVA for colorectal cancer: HTC-116**

| *Source* | *Sum of squares* | *FD* | *Medium squared* | *F-value* | *P-value* |
|---|---|---|---|---|---|
| Between sample means | 36,5616 | 29 | 1,26074 | 354,66 | 0,0000 |
| Within samples | 0,568768 | 160 | 0,0035548 | | |
| Total | 37,1303 | 189 | | | |

**ANOVA for fibroblasts: HS-5**

| *Source* | *Sum of squares* | *FD* | *Medium squared* | *F-value* | *P-value* |
|---|---|---|---|---|---|
| Between sample means | 15,0786 | 29 | 0,51995 | 437,91 | 0,0000 |
| Within samples | 0,191164 | 161 | 0,00118735 | | |
| Total | 15,2697 | 190 | | | |

**ANOVA for liver cancer: HepG2**

| *Source* | *Sum of squares* | *FD* | *Medium squared* | *F-value* | *P-value* |
|---|---|---|---|---|---|
| Between sample means | 8,04467 | 29 | 0,277403 | 316,06 | 0,0000 |
| Within samples | 0,140431 | 160 | 0,000877694 | | |
| Total | 8,18511 | 189 | | | |

**ANOVA for breast cancer: BT474**

| *Source* | *Sum of squares* | *FD* | *Medium squared* | *F-value* | *P-value* |
|---|---|---|---|---|---|
| Between sample means | 35,6583 | 29 | 1,2296 | 426,95 | 0,0000 |
| Within samples | 0,463674 | 161 | 0,00287996 | | |
| Total | 36,122 | 190 | | | |

## Supplementary material S5

## Cell cycle phase analysis

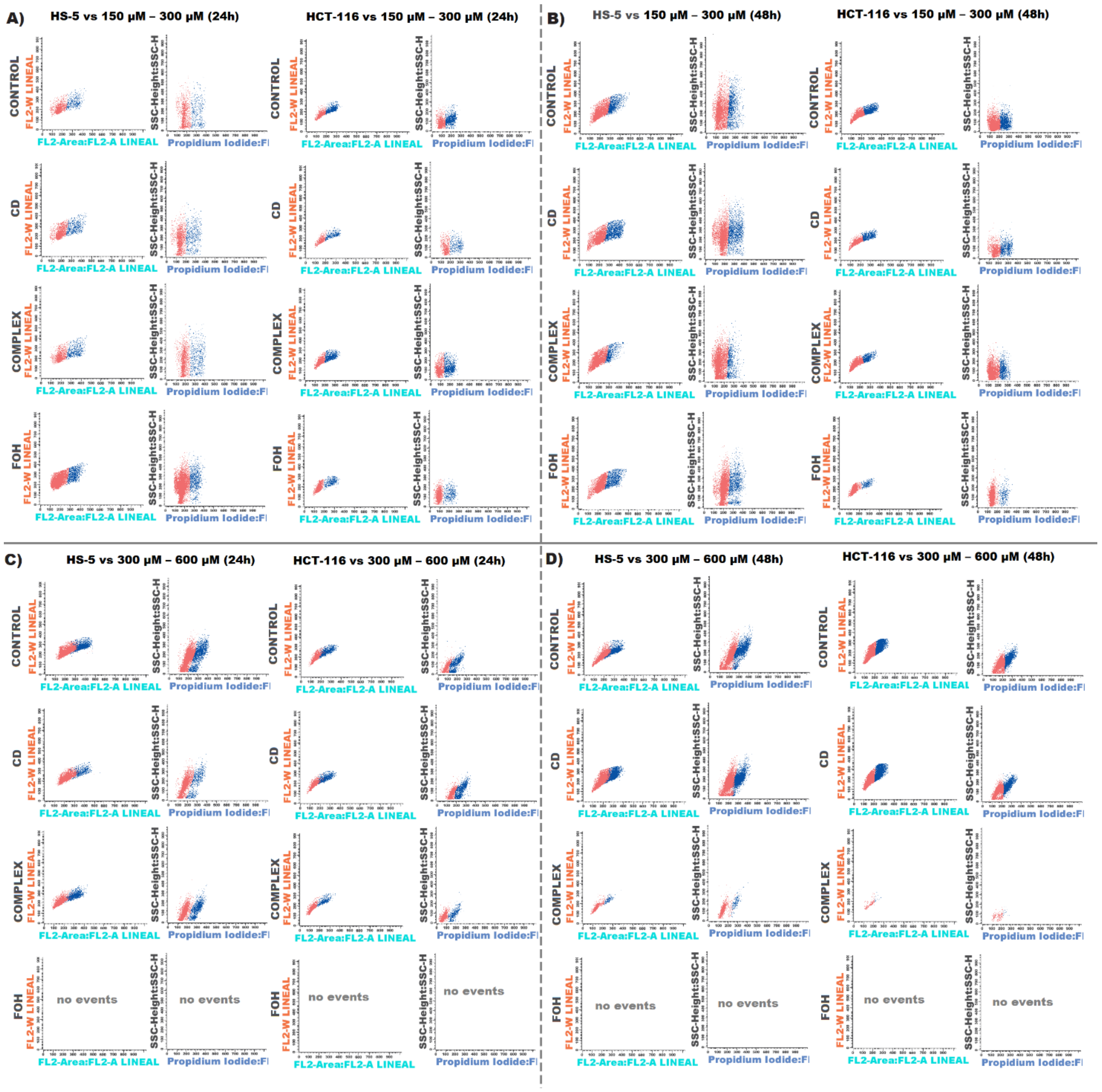


*Figure S12: Graphics of cells in G0/G1 (red) or M/G2 (blue) phase after treatment FOH 150 µM and SBE-β-CD 300 µM of fibroblast and colorectal cancer cells for 24 (A) and 48 hours (B), or after treatment FOH 300 µM and SBE-β-CD 600 µM cells for 24 h (A) and 48 h (B).*
